# Poster Session II - A252 CLINICAL CHARACTERISTICS OF INFLAMMATORY BOWEL DISEASE IN PATIENTS WITH CHRONIC NONBACTERIAL OSTEOMYELITIS: A CASE SERIES

**DOI:** 10.1093/jcag/gwaf042.252

**Published:** 2026-02-13

**Authors:** M Hussain, S Benabess, A Binaqail, R Scuccimarri, N Ahmed

**Affiliations:** Department of Pediatrics, McGill University, Montreal, QC, Canada; McGill University Faculty of Medicine and Health Sciences, Montreal, QC, Canada; Department of Pediatrics, McGill University, Montreal, QC, Canada; Department of Pediatrics, McGill University, Montreal, QC, Canada; Department of Pediatrics, McGill University, Montreal, QC, Canada

## Abstract

**Background:**

Chronic nonbacterial osteomyelitis (CNO) is a rare pediatric autoinflammatory bone disorder characterized by relapsing aseptic inflammation. CNO is increasingly recognized as an extraintestinal manifestation of inflammatory bowel disease (IBD), most commonly Crohn’s disease (CD)

**Aims:**

To describe the clinical features, management and outcomes of patients with concomitant CNO and IBD at the Montreal Children’s Hospital.

**Methods:**

We conducted a retrospective chart review of patients <18 years of age at our centre diagnosed with CNO between 2019-2025 and identified those with co-existing IBD. Data was collected on time of diagnosis, clinical and laboratory characteristics and treatment

**Results:**

We identified 31 patients with CNO and among these 4 (13%) had both CNO and IBD. The cohort included 2 males and 2 females. The mean age at CNO diagnosis was 10.5 years (range 9–13), and the mean age at IBD diagnosis was 12.3 years (range 11–14). The interval between both diagnoses ranged from 2 months to 5 years (mean 2.3 years). CD was the predominant phenotype (3/4 patients), while one case was classified as IBD-U. Paris classification among CD patients included L3 and L4b phenotypes. Three out of 4 patients were asymptomatic at the time of diagnosis of IBD, while one had symptoms of abdominal pain and diarrhea.

The number of CNO lesions per patient ranged from 3 to 14 (mean 7.75, SD 4.6). Lesions most frequently involved the pelvic girdle and lower extremities. Laboratory data showed mild anemia in half of the patients (mean Hb 120.5 g/L, range 111–133), with elevated inflammatory markers (ESR, CRP) in only 2 patients. Fecal calprotectin levels were markedly elevated in all tested patients (range 507–2100 µg/g). Initial CNO therapy consisted of nonsteroidal anti-inflammatory drugs (naproxen) in three patients, and infliximab (as part of IBD management) in one. Three of 4 patients were started directly on a biologic (adalimumab or infliximab) for treatment of their IBD while one patient was treated initially with mesalamine and methotrexate and was subsequently escalated to an anti-TNF. One patient required a change to ustekinumab due to refractory GI tract inflammation in the absence of clinical symptoms. At last follow-up, 3/4 (75%) achieved CNO remission, whereas IBD remained active in 3/4 (75%)

**Conclusions:**

This study illustrates the importance of considering a diagnosis of IBD in patients with CNO even in the absence of clinical symptoms. Furthermore, therapeutic responses differ, with anti-TNF agents more consistently achieving bone remission than intestinal control in this cohort. Larger studies are needed to better understand the links between these conditions and to optimize therapeutic strategies

**Funding Agencies:**

None

